# Biofilm Formation Reducing Properties of Manuka Honey and Propolis in *Proteus mirabilis* Rods Isolated from Chronic Wounds

**DOI:** 10.3390/microorganisms8111823

**Published:** 2020-11-19

**Authors:** Joanna Kwiecińska-Piróg, Jana Przekwas, Michał Majkut, Krzysztof Skowron, Eugenia Gospodarek-Komkowska

**Affiliations:** 1 Department of Microbiology, Ludwik Rydygier Collegium Medicum in Bydgoszcz, Nicolaus Copernicus University in Torun, 9 Maria Curie-Skłodowska St., 85-094 Bydgoszcz, Poland; j.kwiecinska@cm.umk.pl (J.K.-P.); skowron238@wp.pl (K.S.); gospodareke@cm.umk.pl (E.G.-K.); 2 Department of Zoology and Landscaping, University of Science and Technology in Bydgoszcz, 28 Mazowiecka St., 85-084 Bydgoszcz, Poland; whibz@utp.edu.pl

**Keywords:** anti-infective agents, apitherapy, biofilms, diabetic foot ulcer, pressure lesion

## Abstract

Chronic wound infections are difficult to manage because of the biofilm formation in the wound environment. New measures for eliminating infections are necessary to increase the chance of wound healing. Apitherapy may be the new solution. The aim of this study was to assess the prevalence of wound infection factors and to examine the impact of Manuka honey and ethanol extract of propolis on biofilm formation of *Proteus mirabilis* isolated from chronic wound infections. According to the findings, the most frequent factors of infection are *Staphylococcus aureus* (46.1%), *Pseudomonas aeruginosa* (35.0%), and *Proteus mirabilis* (10.6%). Minimal inhibitory concentration and minimal bactericidal concentration values were assigned using the microbroth dilution test according to the Clinical and Laboratory Standards Institute. Biofilm of *Proteus mirabilis* isolates was formed in 96-well polystyrene plates and treated with Manuka honey (concentrations from 1.88% to 30.0%) and ethanol extract of propolis (1.0% to 40.0%). After 24 h, the biofilm viability was expressed by formazan absorbance (λ = 470 nm). Manuka honey reduced the biofilm viability in all, and ethanol extract of propolis in most, of the concentrations tested. Ethanol extract of propolis at the concentrations of 20.0% and 40.0%, reduced biofilm viability stronger than ethanol itself. With these results comes the conclusion that these substances can reduce biofilm formation.

## 1. Introduction

The definition of a chronic wound is not clearly outlined in the literature or clinical practice. Chronic wounds do not heal in a standard time because the healing process does not progress. According to literature reports, a wound is presumed chronic after two to eight weeks of a prolonged healing process [1,2,3,4].

In this paper, we focused on chronic wounds related to diabetes (diabetic foot ulcers) and pressure lesions. During their lifetime, 12.0 to 25.0% of diabetic patients will develop ulcerations. An infection will occur in up to 10.0% of cases [5]. Patients suffering from pressure lesions total from 2.0% to 25.0% of hospitalized patients [4].

The occurrence of planktonic bacteria or even biofilm in the wound is not sufficient to diagnose the infection. Exhibiting a cut-off value (10^6^ colony-forming units (CFU) per gram of tissue sample) in the microbiological examination is not enough without manifested symptoms [5]. According to Delphi [6], highly specific symptoms of chronic wound infection are: cellulitis, acute and progressive course of infection, reddening, localized pain, raised warmth, swelling, tenderness, lymphatic vessels infection, phlegmon, suppuration, or abscess. 

Bacteria species that most frequently cause diabetic foot ulcer infection are *S. aureus*, streptococci group A and B, *Enterococcus* spp., Gram-negative Enterobacterales, and *P. aeruginosa*. Chronic infections are frequently complicated and have mixed etiology [7]. According to the literature data, *Proteus mirabilis* causes about 4.0% of diabetic foot ulcer infections and 9.0% to 13.0% of lesion infections [8,9]. 

*P. mirabilis* is a Gram-negative rod, a component of human and animal gut microbiota. It is the most common ethological agent of infections among *Proteus* species [10]. Among other virulence factors, *P. mirabilis* has a strong ability to form biofilm [10]. 

The biofilm occurs in most (75.0%) chronic wounds, constricting and prolonging the wound healing process. It can be infection causative, although its presence is not equivalent to infection. Biofilm prolongs granulation and raises bacteria resistance to antiseptics and antimicrobials [11]. 

Topical antibiotics are not recommended in infected chronic wound therapy. Systemic use of antibiotics is justified only for antibiotics with high bioavailability. Some antiseptics are toxic for bacteria and also for eukaryote cells, disturbing the process of wound healing [5]. This is the reason why researchers seek other bioactive substances to use in infected chronic wounds. A rich source of many substances exhibiting antimicrobial activity is apitherapy. In this study, we investigated two bee-derived products from New Zealand, Manuka honey (MH) and propolis.

Manuka honey is produced by bees from *Leptospermum scopiarum* flowers in New Zealand. The antibacterial activity of MH depends on several factors. Some factors, such as carbohydrates content, high osmolarity, and low pH level (about 3.9) are common for every type of honey, but others, such as methylglyoxal (MGO) or leptosine, are specific only for MH [12,13,14,15,16,17]. In addition, the high content of phenols and flavonoids and lack of hydrogen peroxide (correlated with a high content of MGO) distinguish MH [16,17,18,19,20,21,22].

Unique Manuka factor (UMF) represents the antibacterial activity of honey in phenol concentration causing the same *S. aureus* growth inhibition zone in agar plates [23]. UMF is correlated with MGO content in MH [19,24]. 

Propolis or bee glue is a bee-derived product made of raisin and beeswax. It is used to seal the beehive and protect it from bacteria and fungi. Propolis consists of 50% raisin (triterpenes), 30% wax, 10% essential oils, 5% pollen, and other organic substances. Flavonoids such as pinocembrin, chrisin, and pinobanksin are the main factors known for their antioxidant, antivirus, and antibacterial activity [25,26]. Pinocembrin and pinobanksin are dihydroflavonoids. Most of the flavonoids in New Zealand propolis are dihydroflavonoids (70.0%), which is respectively high [27]. This may indicate differences in antibacterial activity among propolis of different origins. 

There are a few products on the market available, for example, for wound dressings that contain bee-derived products. Medihoney^®^ is a medical-grade MH used for acute, chronic, or surgical wounds and superficial burns for autolytic debridement promotion or malodor reduction. Although Medihoney^®^ is promoted as a dressing for infected wounds, the producer does not claim that it has proven antimicrobial activity.

## 2. Materials and Methods 

### 2.1. Prevalence of P. mirabilis in Wound Infections of Chronic Wounds at Dr. Antoni Jurasz University Hospital No. 1 in Bydgoszcz from 2016 to 2018

To examine the epidemiology of infected chronic wounds at University Hospital No. 1 in Bydgoszcz, Poland, from 2016 to 2018, we evolved epidemiology data. All positive results of chronic wound examinations (contamination excluded), i.e., a total of 1142, were included in the examination. We did not include anaerobes in the present paper.

### 2.2. Proteus Mirabilis Examined Clinical Strains 

Isolates (*n* = 31) were obtained from the clinical isolates collection of the Clinical Microbiology Department of Dr. Antoni Jurasz University Hospital No. 1 in Bydgoszcz. The strains were chosen randomly from chronic wound infection isolates collected through the years 2016–2018. Strains were stored in brain-heart infusion broth (BHI, Becton Dickinson, Franklin Lakes, NJ, USA) with 20.0% glycerol (Avantor, Gliwice, Poland), at −70 °C. 

### 2.3. Reference Strains

Strain selection was based on the PN-EN 1040:2006E standard quantitative dilute method for bactericidal activity of disinfectants and antiseptics (Appendix A, Table A1). The following six reference strains were obtained from the American Type Culture Collection (ATCC^®^): *Escherichia coli* ATCC^®^ 25922™ (ECO 25922™), *E. coli* ATCC^®^ 35218™ (ECO 35218™), *Enterococcus faecium* ATCC^®^ 29212™ (EFA 29212™), *S. aureus* ATCC^®^ 25923™ (SAU 25923™), *S. aureus* ATCC^®^ 29213™ (SAU 29213™), and *P. aeruginosa* ATCC^®^ 27853™ (PAE 27853™). 

### 2.4. Manuka Honey

Manuka honey was obtained from Manuka Health, New Zealand and stored at room temperature in the dark. The producer ensures that the honey MGO index is 400+ (at least 400 mg of MGO in 1 kg of honey). Honey sterility assay was conducted using a Tryptic Soy Agar plate (TSA, Becton Dickinson, Franklin Lakes, NJ, USA) with the addition of MH preparation and incubation at 37 °C for 5 days and checking for any trace of microbe growth. The MH concentrations for the investigation were prepared by a series of dilutions in Tryptic Soy Broth (TSB, Becton Dickinson) to the final concentrations of 1.87%, 3.75%, 7.50%, 15.0%, 20.0%, and 30.0%.

#### 2.4.1. Polyphenols Content in Manuka Honey

The polyphenol content of MH was examined using the Folin–Ciocalteu colorimetric method modified by Keutgen and Pawelzik [28] (λ = 736 nm). The test was taken twice. The obtained mean values were 827.2 mg·kg^−1^ gallic acid equivalents (GAE) and 701.4 mg·kg^−1^ GAE.

#### 2.4.2. Antioxidant Activity of Manuka Honey

The antioxidant activity of MH was tested with the ferric reducing antioxidant power (FRAP) assay. The test was repeated twice. The obtained values were 0.33 and 0.32 mmol·kg^−1^.

### 2.5. New Zealand Propolis Solution Preparation Method

New Zealand propolis, in the dose of 80.0 g, was mixed with 100 mL of ethanol and incubated at room temperature for 14 days. Next, the solution was stored at −20 °C for 30 days for the insoluble compound sedimentation. Only supernatant, ethanol extract of propolis (EEP), was used in the research. The extract was filtered through a sterile syringe (0.22 nm; ChemLand, Stargard, Poland). The mean weight of active substances in 1 mL of EEP was 0.147 g. A series of concentrations of EEP were prepared by the dilution of EEP in TSB to final concentrations of 1.0% (1.5 mg·mL^−1^), 2.50% (3.7 mg·mL^−1^), 5.0% (7.4 mg·mL^−1^), 10.0% (14.7 mg·mL^−1^), 20.0% (29.4 mg·mL^−1^), and 40.0% (58.8 mg·mL^−1^).

### 2.6. Minimal Bactericidal Concentration and Biofilm Bactericidal Concentration of Manuka Honey and New Zealand Propolis in Reference Strains

Due to the precipitation of insoluble components of honey and propolis, reliable minimal inhibitory concentration (MIC) values were impossible to obtain. For this reason, the assessment of minimal bactericidal concentration (MBC) was chosen.

The assessment of MBC for planktonic cells was based on the Clinical and Laboratory Standard Institute (CLSI) recommendation standard. MBC and biofilm bactericidal concentration (BBC) for a 24-h mature biofilm (0.5 McFarland standard inoculum undiluted and diluted 1:100) were assigned for reference strains (Section 2.3). The well content was absorbed for MBC and bottoms of wells were scraped for BBC. Next, the well content was seeded on the TSA plate.

### 2.7. Minimal Biofilm Inhibitory Concentration, Biofilm Prevention Concentration, and Minimal Biofilm Eradication Concentration of Manuka Honey and New Zealand Propolis in Clinical Strains

Minimal biofilm inhibitory concentration (MBIC), biofilm prevention concentration (BPC), and minimal biofilm eradication concentration (MBEC) [29], for approximately $1.5\times 10^{9}$ CFU·mL^−1^ inoculum, were assigned for the *P. mirabilis* clinical strains. For each strain, the suspension control was performed by seeding aliquots of the positive control well on CLED agar and CFU·mL^−1^ counting after 24-h incubation.

MBIC and BPC were assigned by simultaneous bacterial inoculation and substances exposure, and MBEC was tested on the 24-h mature biofilm. After the final incubation, MBIC was read by the absence of opacity, while BPC and MBEC were read as the lowest concentration preventing growth after seeding well content on the CLED agar plate.

### 2.8. Impact of Manuka Honey and Ethanol Extract of Propolis (EEP) on the Metabolic Activity of P. mirabilis Mature Biofilm 

#### 2.8.1. Biofilm Cultivation

Bacteria inocula (0.5 McFarland optic density, see Appendix A, Table A1) were prepared in the TSB from the 24-h mature CLED agar cultures. Inocula and TSB were added to each well in 1:1 (*v/v*) proportion. Inocula in TSB (0.5 McFarland optic density) were added directly to the wells of polystyrene plate or were primarily diluted in sterile TSB (1:100). Negative control wells proved that the suspension media were sterile. Plates were covered with lids and incubated in a humid atmosphere at 37 °C. After 24 h, the suspension medium with planktonic cells was removed and wells were washed thrice with PBS (phosphate buffer saline) (pH 7.2).

#### 2.8.2. Manuka Honey and EEP Impact on Biofilm

To examine the impact of MH and EEP on the biofilm, serial dilutions of substances in liquid medium were added to wells containing the washed biofilm. Plates were covered with lids and incubated in a humid chamber at 37 °C, for 24 h. To compare the impact of EEP on the mature biofilm formation with ethanol, ethanol solution in PBS (1.25:1 *v/v*) was simultaneously tested. For each strain, the impact of all concentrations was determined in triplicate. 

#### 2.8.3. Assessment of Mature Biofilm Metabolic Activity after 24 h of Manuka Honey or EEP Treatment

To assess the metabolic activity of biofilm cells, polystyrene plates (containing biofilm treated with the solutions tested) were rinsed with PBS, then filled with 1% (*w/v*) 2,3,5-triphenyl-2H-tetrazolium chloride (TTC, Avantor, Gliwice, Poland) in TSB (1:9 *v/v*), and incubated in humid conditions at 37 °C. After 4 h, the supernatant was removed, wells were rinsed with water, and filled with methanol (Avantor). The absorbance of formazan was read at 470 nm (Synergy, BioTek, Winooski, VT, USA).

### 2.9. Statistical Analysis

Epidemiologic data were analyzed using Excel 2007.

The grade of metabolic activity reduction of strains was calculated in Excel 2007 based on the equation:

Metabolic activity = [A (K+) − A (x)]/[A (K+)]
(1)

where:

- A (K+) means positive control absorbance;
- A (x) means absorbance after treatment with honey or EEP in defined concentration.

Statistical analysis was performed using Statistica 13.1. The assessment of independent variables was conducted using Wilcoxon’s test. The null hypothesis was stated as, “Mean grades of metabolic activity after MH, ethanol extract of propolis, and ethanol treatment in all examined concentrations are not statistically different.” The statistical significance (P) was determined at level 0.05. Independent variables were considered to be statistically significant at *p* < 0.05.

## 3. Results

### 3.1. Prevalence of P. mirabilis in Wound Infections of Chronic Wounds at Dr. Antoni Jurasz University Hospital No. 1 in Bydgoszcz from 2016 to 2018

*P. mirabilis* is third among the most frequently isolated species (10.6%). Although it is not as an emergent pathogen as multidrug-resistant *Klebsiella pneumoniae* or *Acinetobacter baumannii*, its ability to form biofilm in wound environment allows *P. mirabilis* to survive and be infection causative. The most frequent factors isolated from chronic wounds are *S. aureus* (isolated from 46.1% of cases) and *P. aeruginosa* (35.0%). 

Among all cases of chronic wound infections, there are 285 (25.0%) monocultures and 857 (75.0%) mixed cultures. The most frequent factor of infection in monocultures is *S. aureus* (51.2%), the second most frequent is *P. aeruginosa* (28.4%), and the third most frequent is *P. mirabilis* (4.2%). 

Infection is the result of mixed cultures in 857 (75.0%) cases. The most frequent tandems of microorganisms are *S. aureus* coexisting with *P. aeruginosa* (12.5%), and *P. aeruginosa* coexisting with *P. mirabilis* (5.9%). Moderately frequent tandems are *S. aureus* coexisting with *Enterobacter cloacae*, *P. mirabilis*, *Streptococcus agalactiae*, or *Streptococcus dysgalactiae*. These pairs occur in 3 to 5% of cases. 

Generally, more than two microorganisms are isolated in 498 cases, which is 58.1% of all chronic wound infections. The bacteria most frequently isolated as accompanying microbiota are *Enterococcus* spp. (33.7% of all cases). Coagulase-negative *Staphylococcus* spp. and *Corynebacterium* spp. strains found in insignificant quantities are treated as skin microflora contamination and occurred in 14.7% of all cases.

### 3.2. Comparison of Minimal Bactericidal Concentration (MBC) and Biofilm Bactericidal Concentration (BBC) Values of Manuka Honey and EEP in Reference Strains

A comparison of MBC and BBC values for six reference strains after MH and EEP treatments is presented in Table 1. 

Lower values of MBC for EEP as compared with MH indicated that EEP suppressed the growth of planktonic cells more effectively. For 24-h mature biofilm cultured from 0.5 McFarland standard, the BBC values of MH are lower than EEP in four out of six strains, while for 1:100 (*v:v*) dilution, the BBC values of MH are lower than EEP in five out of six strains. As for EEP, the MBC values are always lower than BBC values. There is no regularity for MH.

### 3.3. Comparison of MBIC, BPC, and MBEC Values of Manuka Honey and EEP in Proteus Mirabilis Clinical Strains

The MBIC, BPC, and MBEC values of the examined solutions are obtained for 0.5 McFarland suspensions. The results are presented in Table 2 and Table 3.

The MBIC values for MH visually fluctuate within a range from 20.0% to 30.0%, but BPC values ascend to 30.0% and higher. The MBEC values of the mature biofilm in all examined strains stand at more than 30.0%.

The MBIC value of EEP is situated in a range from below 1.0% to 5.0%, while most strains obtain 2.5% value. The MBIC value of ethanol has a higher range (from 2.5% to 10.0%) and most strains obtain the value 5.0% (Table 4). BPC shows no distinguishable difference, values are mainly distributed evenly between 5.0% and 10.0% concentration for EEP and ethanol. For EEP, the range values of MBEC are from 5.0% to 20.0%. Most strains reach the MBEC value of 10.0%. For ethanol, there is a much wider range, i.e., from 1.0% to more than 40.0%. Nine strains reach the MBEC of 40.0% ethanol or more. Ten strains reach an MBEC value of 2.5% ethanol or less. 

The noticeable tendency of increasing the obtained values arranged in Table 2 and Table 3 is preserved except for the last column in Table 3, where the obtained MBEC of ethanol for mature biofilm has a wide range, while all other ranges are narrowed. However, this tendency confirms that cells arranged in a biofilm are more resistant than planktonic forms.

### 3.4. Presentation of Causality between Manuka Honey or EEP Impact and Metabolic Activity of the Mature Biofilm Derived from Clinical Strains 

**Figure 1:**
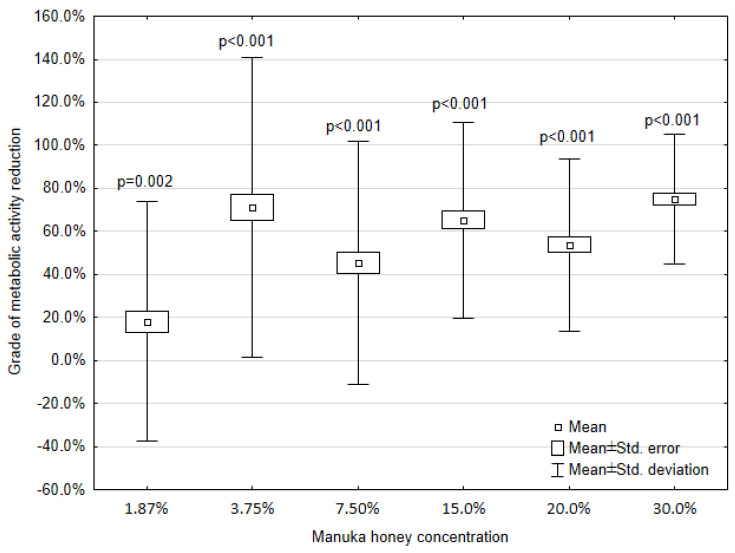
Impact of Manuka honey on the metabolic activity of *P. mirabilis* biofilm; *p*-values refer to the comparison between MH concentration and the positive control.

**Figure 2:**
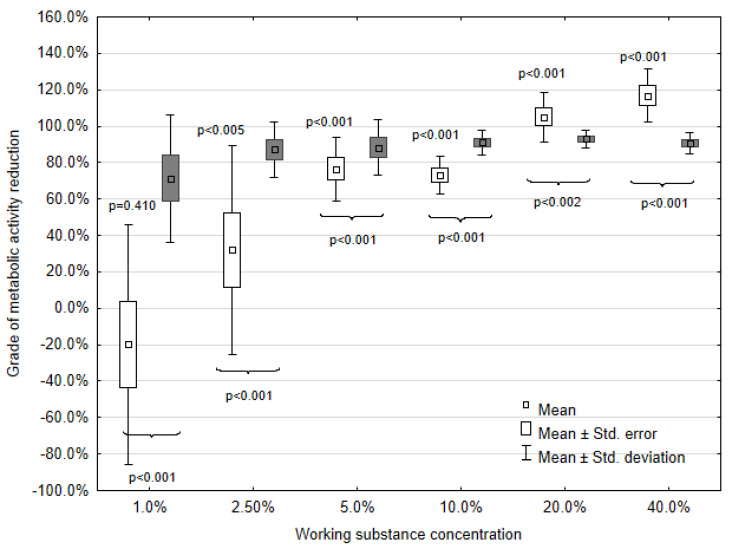
Impact of ethanol extract of propolis (EEP, white color) and ethanol (EtOH, grey color) on the metabolic activity in *P. mirabilis*; *p*-values on the boxplot above whiskers refer to the EEP and positive control comparison; *p*-values below whiskers refer to the EEP and EtOH comparison.

Statistical analysis of the obtained data was calculated as described above. Because of the absorbance adjustment for propolis, the percentage values are above 100%. Values below zero prove the acquisition of strain metabolic activity as compared with the positive control. To exhibit the causality between MH or EEP impact and metabolic activity of the mature biofilm derived from clinical strains (*n* = 31), the Wilcoxon signed rank tests were taken. The results of the statistical analysis of metabolic activity are presented in Figure 1 and Figure 2. 

The loss of absorbance after each of the MH or EEP concentration treatments as compared with the positive control is manifested in grades of metabolic activity reduction. As expected, the highest mean grade of metabolic activity reduction is reached for 30.0% concentration of MH (75.0%) and 40.0% concentration of EEP (116.9%). In the concentration range from 40.0% to 5.0%, EEP causes a higher reduction of metabolic activity than MH in the concentration range from 30.0% to 7.50%. EEP concentrations lower than 5.0% result in a lower mean metabolic activity reduction than MH. After the treatment with the lowest concentrations (1.88% for MH and 1.0% for EEP), MH causes a mean metabolic activity reduction of 18.0%, while EEP causes a mean metabolic activity increase of 19.0%. 

With a decrease in the MH or EEP concentration, an increase in absorbance and a lower level of metabolic activity reduction is visible, except for the absorbance after 3.75% MH treatment. A clear increase in the level of metabolic activity reduction is present as compared with the concentration ranges 7.5%, 15.0%, and 20.0%. 

Probability values below 0.05 and even 0.01 indicate the presence of statistically significant differences between the absorbances obtained for each MH concentration and the positive control (0.0%).

The probability values, obtained for the EEP concentration range from 2.5% to 40.0%, are below 0.05. This indicates that there is a statistically significant difference between the absorbance obtained after EEP treatment (in this range of concentrations) and absorbance obtained with the positive control (0.0%). Only with a concentration of 1.0%, the probability value was above 0.05.

### 3.5. Comparison of the EEP and EtOH Impact 

To compare the EEP and ethanol (EtOH) impact on the absorbance depicting the metabolic activity of clinical strains, the Wilcoxon signed rank test was taken. The results of the statistical analysis of data are presented in Figure 2. 

Only after EEP treatment with concentrations of 40.0% and 20.0%, the level of metabolic activity reduction is higher as compared with EtOH. The mean grades of metabolic activity reduction for EEP are 116.9% and 105.0%, respectively, and 90.7% or 93.1% for EtOH. As for the concentration range from 10.0% to 1.00%, the mean grade of metabolic activity reduction for EEP is consistently lower than for EtOH. The mean grades of metabolic activity reduction values for 10.0% to 1.0% EEP are 73.2%, 76.4%, 32.1%, and −19.0%, respectively, while for EtOH, the values are 91.1%, 88.3%, 87.3%, and 71.3%.

Statistically significant differences between absorbance values were proven for each tallied pair of EEP and EtOH concentrations (Figure 2).

## 4. Discussion

Bacteriology of chronic wound infections is being constantly monitored by scientist and clinicians all over the world, and the vast majority of articles have reported that the most prevalent bacteria isolated from chronic wounds were *S. aureus*, *S. epidermidis*, and other coagulase-negative staphylococci, as well as *Streptococcus* spp., *P. aeruginosa*, and other Gram-negative rods [9,30,31,32].

In the bacterial profile described in this paper, the most frequently isolated bacteria were *S. aureus* (46.1%), *P. aeruginosa* (35.0%), and *P. mirabilis* (10.6%). In most cases (75.0%), chronic wound infections had a mixed etiology. Most infections were caused by tandems of *S. aureus* and *P. aeruginosa* (12.5%) or *P. aeruginosa* and *P. mirabilis* (5.9%). Mixed etiology of the infection hindered antibiotics penetration to the wound environment, and defective flow through the venous system altogether obstruct the chronic wound healing and decrease the quality of life of patients. Apitherapy products incorporated into dressings may fasten the healing process and attenuate the infection.

Several research groups have attempted to verify the antimicrobial effect of Manuka honey and propolis [17,33,34,35]. Maddocks S. et al. [36] proved that Manuka honey inhibited *S. aureus*, *P. aeruginosa*, and *S. pyogenes* bacterial cells in binding to the human proteins, i.e., fibronectin, fibrinogen, and collagen. Manuka honey treatment also caused a reduction in binding to human keratinocytes by all these species and bacterial invasion of *S. pyogenes* spp. A publication by Lu J. et al. [35] presented the impact of Manuka honey and Medihoney^®^ on the *S. aureus* biofilm using a CV assay. Their results presented a significant decrease of the biofilm biomass in all the strains tested after treatment with honey concentrations ranging from 16.0% to 32.0%. Generally, the metabolic activity of strains decreased proportionally to the biomass. Results of the present experiment confirm that Manuka honey affects the metabolic activity of *Proteus mirabilis* strains. Emineke S. et al. [37] reported that Manuka honey treatment of established bacterial biofilms of *P. mirabilis* and *E. coli* was most effective after 24-h incubation; the most abundant biofilm reduction was shown after 24 h as compared with 4 h of honey treatment, and early biofilm formation was inhibited even at a concentration as low as 3.3% if the exposure was long enough (72 h in this case).

In this research, following our expectations, Manuka honey MBIC and MBEC values of *P. mirabilis* clinical strains were slightly higher than MIC or MBC values obtained in other papers [38,39,40]. However, although the MIC and MBC microdilution broth method is widely used, the MBIC, MBEC, or BPC methods are not standardized; these values can differ depending on the experimental conditions. As for the reference strains, values obtained for SAU 25923™, ECO 25922™, ECO 35218™, and PAE 27853™ are comparable to those obtained by Tze H. T. et al. [38] and Sherlock O. et al. [41] (Table 4). Table 4 indicates that the unique Manuka factor, which reflects the methylglyoxal content in honey, is connected to the antibacterial effect of Manuka honey. Biofilm bactericidal concentration values of Manuka honey obtained in this paper are lower than MBC. This was evident in four out of six reference strains, i.e., SAU 25923™, ECO 25922™, ECO 35218™, and PAE 27853™. This may indicate that Manuka honey effectively inhibits early biofilm formation. Many antibacterial factors contribute to this effect. Methylglyoxal and osmotic stress disrupt bacterial cell walls, hydrogen peroxide induces oxidative stress, while enzymes and peptides produced by bees may act as an antibacterial [42].

There have been a few attempts to evaluate the effectiveness of Manuka honey incorporated in wound dressings against *P. aeruginosa*, *S. aureus*, *E. coli*, and group B Streptococci. Materials used in those studies were cryogel [43] or bioactive glass [44] fortified with Manuka honey and bacterial cellulose enriched with methylglyoxal [45]. However, some of these dressings manifested antimicrobial effects too weak to be used. A few research groups throughout the years 2014–2018 have attempted to study the effectiveness of Manuka honey in wound healing. Most of the results have indicated that Manuka honey had a positive effect on wound healing, shortening the complete time needed for ulcer healing. However, these results need to be treated with caution due to the limited data on patients, baseline characteristics of ulcers, and poor study design [46]. Manuka honey is also used in the therapy of other conditions, such as chronic rhinosinusitis and evaporative dry eye. Randomized controlled trials were performed by Albietz J. et al. [47] and Lee V.S. et al. [48].

Several studies have reported MIC or MBC values of propolis from different sources (red propolis, geopropolis, and many others) obtained for *S. aureus*, *S. mutans* [49,50], oral Streptococci, and *E. faecalis* [51]. Kouidhi et al. [51] obtained a MIC value above 0.512 mg·mL^−1^ for EFA 29212™ as compared with 7.4 mg·mL^−1^ in this research. The MBC value for SAU 25923™ was 7.4 mg·mL^−1^ in the present study, while in 2007 [49], it was 0.20–0.40 mg·mL^−1^ and, in 2013 [50], it was 0.3130 mg·mL^−1^. This may be the result of differences in the composition of propolis used in the assay.

In 2018, de Oliveira Dembogurski D.S. et al. [52] conducted an assessment of the impact of Brazilian propolis on the metabolic activity of *S. aureus* biofilm cultured for 24 h. Propolis derived from two sources decreased the metabolic activity of the biofilm, but with propolis from the third source, a significant difference was noticeable only after treatment with the concentration of 2 mg·mL^−1^. In this research, the lowest concentration of EEP that caused a significant difference was 3.7 mg·mL^−1^ (2.50%).

Cases of treatment with ethanol extract of propolis have been described in the scientific literature since 1969. Ethanol extract of propolis has been used with chronic wounds, ulcers, surgical site infections, and burns, but also in cases of vaginitis or dental sockets [53,54,55,56,57]. Alongside its antibacterial, antiviral, and antifungal properties, due to the high content of flavonoids [24,25], it also promotes wound healing. The presence of caffeic acid phenyl ester provides antiproliferative activity against human colorectal adenocarcinoma cells [58], antioxidative activity, and promotes re-epithelization and wound closure in mice pressure ulcers [59,60], which may be helpful in chronic wound infection when the immune system is not efficient enough to fight the biofilm and prolonging inflammation leads to tissue damage.

Oral uptake of propolis alone and in combination with silver nanoparticles increased fibroblast proliferation, collagen deposition, and provided an anti-inflammatory effect on experimental wounds in rats [61]. There were also numerous attempts to design wound dressings with incorporated propolis. The most promising material for this application is chitosan. Chitosan-propolis nanoparticles have been proven to have anti-biofilm activity against *E. faecalis* [62]. Chitosan-based coacervates prepared for propolis encapsulation have been reported to be non-cytotoxic [63]. Another study proved that propolis and essential oils incorporated in biocellulose membranes exhibited antibacterial and proinflammatory activity, and improved wound healing in rats [64]. Other materials used for propolis incorporation are corn starch dressings [65] and polyurethane-hyaluronic acid nanofibrous dressings [66].

Antimicrobial activities of Manuka honey and propolis depend on their concentration, MGO content, polyphenols content, antioxidant activity, high osmolarity, and several other factors. However, their antimicrobial activity is incontestable. Manuka honey suppresses the metabolic activity of *P. mirabilis* biofilm in all examined concentrations (1.88% to 30.0%). Ethanol extract of propolis suppresses the metabolic activity of *P. mirabilis* more tenuously than ethanol in concentrations beneath 20.0% (29.4 mg·mL^−1^). 

Further investigation should focus on the selection of the best material for wound dressing enriched with propolis or Manuka honey. The material used for dressing preparation should be a fine carrier for antibacterial substances and air permeable, but should also provide high humidity for the wound environment and be easy to remove. 

## Figures and Tables

**Table 1:** Minimal bactericidal concentration (MBC) and biofilm bactericidal concentration (BBC) values of Manuka honey (MH) and ethanol extract of propolis (EEP) obtained with reference strains.

Reference Strains	MH (*v/v*%)			EEP (*v/v*%)		
	MBC	>BBC		MBC	>BBC	
		0.5 MF	1:100 Dilution ^a^		0.5 MF	1:100 Dilution ^a^
*SAU 25923™*	20.0	20.0	15.0	5.0	20.0	40.0
*SAU 29213™*	20.0	25.0	25.0	5.0	40.0	40.0
*EFA 29212™*	20.0	25.0	25.0	5.0	20.0	20.0
*ECO 25922™*	15.0	10.0	10.0	5.0	40.0	40.0
*ECO 35218™*	15.0	10.0	10.0	5.0	20.0	40.0
*PAE 27853™*	25.0	>25.0	15.0	10.0	40.0	40.0

^a^ 1:100 (*v:v*) dilution of 0.5 McFarland standard inoculum in Tryptic Soy Agar.

**Table 2:** Comparison of *Proteus mirabilis* ^a^ susceptibility to Manuka honey (MH).

MH Concentration (*v/v*%)	Number of *P. mirabilis* Strains (%)		
	MBIC ^b^ 0 h	BPC ^c^ 0 h	MBEC ^c^ 24 h
>30.0	0 (0.0)	11 (35.5)	31 (100.0)
30.0	28 (90.3)	20 (64.5)	0 (0.0)
20.0	3 (9.7)	0 (0.0)	0 (0.0)
15.0	0 (0.0)	0 (0.0)	0 (0.0)
Total number of strains	31 (100.0)	31 (100.0)	31 (100.0)

^a^ MH impact on 1.50 × 10^9^ CFU per mL (0.5 McFarland) inoculum of *P. mirabilis* forming a biofilm (0 h) and mature biofilm (24 h) (*n* = 31). ^b^ MBIC obtained visually by the absence of opacification. ^c^ BPC and MBEC obtained by plating wells content on CLED agar and 24-h incubation at 37 °C.

**Table 3:** Comparison of *Proteus mirabilis* ^a^ susceptibility to ethanol extract of propolis (EEP) and ethanol (EtOH).

C [*v/v*%]	Number of *P. mirabilis* Strains					
	MBIC ^b^ 0 h (%)		BPC ^c^ 0 h (%)		MBEC ^c^ 24 h (%)	
	EEP	EtOH	EEP	EtOH	EEP	EtOH
>40.0%	-	-	-	-	-	3 (9.67)
40.0	-	-	-	-	-	6 (19.3)
20.0	-	-	5 (16.1)	3 (9.7)	3 (9.7)	2 (6.45)
10.0	-	1 (3.2)	12 (38.7)	14 (45.2)	21 (67.7)	6 (19.3)
5.0	4 (12.9)	22 (71.0)	12 (38.7)	13 (41.9)	7 (22.6)	4 (12.9)
2.5	17 (54.8)	8 (25.8)	2 (6.5)	-	-	7 (22.6)
1.0	4 (12.9)	-	-	1 (3.2)	-	3 (9.7)
<1.0	6 (19.4)	-	-	-	-	-
Total number of strains	31 (100.0)					

^a^ EEP and EtOH impact on 1.50 × 10^9^ CFU per mL (0.5 McFarland) inoculum of *P. mirabilis* forming a biofilm (0 h) and mature biofilm (24 h) (*n* = 31). ^b^ MBIC obtained visually by the absence of opacification. ^c^ BPC and MBEC obtained by plating wells content on CLED agar and 24-h incubation at 37 °C.

**Table 4:** Manuka honey minimal inhibitory concentration (MIC) and MBC values obtained for selected bacteria based on literature review and own research.

Manuka Honey			
Strain	UMF 10+ ^a^	UMF 25+ ^b^	Values Maintained in This Study (UMF 13+)
*S. aureus* ATCC^®^ 25923™	MBC 25.0%	-	MBC 20.0%
*P. aeruginosa* ATCC^®^ 27853™	MBC 22.5%	MBC 12.5%	MBC 25.0%
*E. coli* ATCC^®^ 25922™	MBC 15.0%	-	MBC 15.0%
*E. coli* ATCC^®^ 35218™	-	MBC 12.5%	MBC 15.0%

^a^ (Tze H.T. et al., 2009), ^b^ (Sherlock O. et al., 2010).

## Abbreviations

MBIC	Minimal biofilm inhibition concentration, defined as the lowest concentration of a substance that inhibits visible growth of 24-h mature biofilm culture.
BPC	Biofilm prevention concentration, defined as the lowest concentration of a substance that inhibits visible growth in the culture where bacterial inoculation and substance exposure occur at the same time.
MBEC	Minimal biofilm-eradication concentration, defined according to [29].
BBC	Biofilm bactericidal concentration, defined according to [29].

## Appendix A

**Table A1:** Juxtaposition of 0.5 McFarland standard with CFU per mL for reference strains.

Reference Strain	CFU Per mL	Standard Deviation
SAU 25923™	1.57 × 10^10^	±1.26 × 10^9^
SAU 29213™	1.28 × 10^9^	±1.16 × 10^8^
EFA 29212™	5.83 × 10^7^	±5.44 × 10^6^
ECO 25922™	3.33 × 10^9^	±5.18 × 10^8^
ECO 35218™	1.45 × 10^8^	±2.73 × 10^7^
PAE 27853™	1.60 × 10^10^	±1.51 × 10^9^
